# An Inquiry into the Alleged Cerebral Origin of Certain Cases of Paraplegia

**Published:** 1827-05

**Authors:** Thomas Harrison Burder

**Affiliations:** one of the Presidents of the Westminster Medical Society; Formerly President of the Medical Society of Edinburgh; and Late Physician to the Westminster General Dispensary.


### THE LONDON
### Medical and Physical Journal.

No. 339, VOL. LVII.] MAY, 1827. [No. 11, *New Series*.

For many fortunate discoveries in medicine, and for the detection of numerous errors, the world is indebted to the rapid circulation of Monthly Journals; and there never existed any work, to which the Faculty, in Europe and America, were under deeper obligations, than to the Medical and Physical Journal of London, now forming a long, but an invaluable, series.—RUSH.

### ORIGINAL PAPERS,

AND

### CASES OBTAINED FROM PUBLIC INSTITUTIONS AND OTHER AUTHENTIC SOURCES.

### PARAPLEGIA.

*An Inquiry into the alleged Cerebral Origin of certain Cases of Paraplegia.* By THOMAS HARRISON BURDER, M.D. one of the Presidents of the Westminster Medical Society; formerly President of the Medical Society of Edinburgh; and late Physician to the WESTMINSTER GENERAL DISPENSARY.

DURING the last ten years, the subject of Paraplegia has attracted considerable attention. Prior to the appearance of the valuable contributions to the pathology of the brain and spinal chord, in the Edinburgh Medical and Surgical Journal for the years 1818-19, by my learned friend Dr. ABERCROMBIE, and the interesting paper on Paraplegia by the late venerated Dr. BAILLIE, in the sixth volume of the Transactions of the London College of Physicians, paraplegia was generally regarded as dependent exclusively on a morbid condition of the spinal marrow, or its investing membranes. The name, indeed, had been employed by the ancient physicians to express very different states of disease; and it was not until a comparatively late period that the term was restricted to its present acceptation.* There is still, however, considerable uncertainty in the extent of its application; some writers confining the term to a paralytic state of the lower half of the body, while others employ it to designate transverse palsy, at whatever point below the neck such pa-

* See Dr. COOKE's discriminating remarks on the opinions of HIPPOCRATES, ARETÆUS, and GALEN, with reference to Paraplegia, in his Treatise on Nervous Diseases.

ralytic affection commences. In this latter and more extended sense, I shall consider the disease in the present paper.

It may be proper to mention that the following remarks were offered as introductory to a discussion on this subject, in the Westminster Medical Society, at the close of the last year. Particular allusion was necessarily made to the influential opinion of Dr. Baillie. In the course of the debate, a distinguished member of the Society, Dr. Gregory, excited a powerful interest by bringing forward an original paper on the subject, confirmatory of the opinions Dr. Baillie had before published, and which that lamented physician had entrusted to Dr. Gregory's care, with an express injunction to produce it, should those opinions ever be particularly controverted. This interesting document Dr. Gregory has obligingly confided to my custody, while prosecuting the present investigation, authorising me to make such use of it as may appear best calculated to subserve the cause of truth,—that noble cause to which Dr. Gregory's inestimable patron had dedicated his active and honourable life.

As the prevailing opinion relative to the cerebral origin of paraplegia may be principally traced to the writings of Drs. Baillie and Abercrombie, I propose first to present a short analysis of the cases from which those able pathologists appear chiefly to have drawn their inferences; adding a few remarks on what appears to me to be the inconclusive nature of the evidence thus furnished. It will then be proper to introduce the posthumous paper of Dr. Baillie before alluded to, with an inquiry how far the additional illustrations thus obtained serve to establish the author's favourite opinion. I shall afterwards advert, cursorily, to the sentiments of a few other writers on the same subject; and, finally, submit to the profession such general inferences from the facts recorded, and from those which have fallen within my own observation, as may appear to be fairly deducible from our present knowledge of the subject.

### I. Dr. Abercrombie's *Cases.*

These interesting examples appeared in the Edinburgh Medical and Surgical Journal for October 1819, and prior to the publication of the volume which contained Dr. Baillie's view of the same subject. They were not adduced, I apprehend, for the purpose of establishing the cerebral origin of paraplegia, but merely formed part of a most valuable series of illustrations on the morbid anatomy of the brain and spinal marrow. They have, however, materially contributed to strengthen that opinion, having been quoted in our late systematic works as admitted examples of cerebral paraplegia. The following cases form the sixth class of Dr. Abercrombie's arrangement of the organic diseases of the brain, and are placed under the general title of "Symptoms in the Head, with Paraplegia." The first and second cases I shall give in the Doctor's own words.

Case I.—"A boy, aged seven, (for whose case I am indebted to Dr. [the late Professor] Gregory,) in the beginning of the year 1811, received a violent injury of his forehead and nose by a fall. From that time he had headache. After two or three months he became near-sighted. Soon after, his sight became indistinct; and, after four or five months more, this was followed by blindness. About this time he began to be epileptic, and affected with weakness of the lower extremities, which gradually increased to perfect paraplegia. He died in April 1812, after coma of three days, his intellect having continued entire till that time.

"*Dissection.*—A firm, white, flat tumor, like a large bean, lay over the junction of the optic nerves. The ventricles contained twelve ounces of clear fluid. The left lobe of the cerebellum was much indurated, like schirrus; the right lobe was reduced to a mass resembling scrofulous pus."

*Remarks.*—On this case I would observe, that, no mention being made of the state of the spine, it may be presumed that the spinal chord was not examined. We have, therefore, no proof that there was not concomitant disease within the vertebral column itself. In the absence of such proof, no satisfactory conclusion can be formed. It appears to me, however, highly probable that the fluid from the ventricles had insinuated itself into the theca vertebralis, giving rise, by its pressure, to the paraplegic symptoms; which symptoms did not appear until evidences of organic mischief had existed for a considerable time. It cannot, therefore, be considered as a satisfactory example of cerebral paraplegia.

Case II. "(From Morgagni, lxii. 15.) A man, aged forty-eight. *Symptoms.*—Acute headache for a year, followed by paralysis of both lower extremities, the superior extremities being sound. Died suddenly about five months after the commencement of the paraplegia.

"*Dissection.*—The left lobe of the cerebellum was almost entirely schirrous, of a pale flesh colour, and seeming to be composed of numerous small corpuscles closely compacted, without any interstice or any appearance of vessels. A small part only on the upper surface was in a healthy state. The corpus callosum, fornix, and some of the other central parts of the brain, were much softened and broken down.

*Remark.*—In this case, also, the spine is not mentioned: consequently, no positive conclusion can be drawn from it.

The third Case is quoted by Dr. Abercrombie from the eleventh volume of the Edinburgh Journal, p. 470. I give it in an abridged form.

*Symptoms.*—Pain of the head, afterwards fixing chiefly in the occiput, and extending down the neck. Occasional vertigo and sickness. After five months, hemiplegia of the left side; imperfect vision. At length, fits of stupor; blindness of right eye; failure of memory; paraplegia. A fortnight before his death, paralysis of the upper extremities also.

*Dissection.*—On the surface of the pons Varolii, there were two triangular fleshy tumors, united by their apices; the base of the one extending into the right crus cerebri, that of the other into the medulla oblongata. Much effusion under the arachnoid membrane.

*Remarks.*—It is much to be lamented that the post-mortem examination did not extend to the spine, as in all probability traces of inflammation would have been found along the spinal arachnoid, with effusion within the theca. This is a fair inference from the symptoms narrated. From an early period of the disease, the pain extended from the occiput down the neck, and considerable effusion was found under the arachnoid of the cerebellum. I have little doubt but that this pain arose from an extension of arachnoid inflammation along the spinal chord, and that effusion of serum supervened, which, accumulating first below, produced paralysis of the lower limbs, and, gradually ascending within the theca, at length similarly affected the upper extremities also. This case cannot therefore be regarded as furnishing any proof of the cerebral origin of paraplegia.

I shall not cite the fourth Case of Dr. Abercrombie, because it is an example of general and complete paralysis, rather than of paraplegia. It would also be liable to the same exception as the former, the spinal chord not being at all mentioned.

It has already been stated that the above cases were not adduced by my learned friend as satisfactory proofs of cerebral paraplegia, but merely as examples of morbid changes *in cranio,* occurring *with* paraplegia. Aware of Dr. Abercrombie's indefatigable zeal in pathological research, I was desirous of ascertaining whether he had since obtained any more satisfactory information on the subject, especially with regard to the state of the spine in similar cases. From a letter with which I was favoured in December last, in answer to some queries of mine, the Doctor appears to look with more distrust on many of the alleged cases of cerebral paraplegia, and candidly remarks—" The more I see of it (paraplegia), the more perplexed I am with regard to its pathology. I have been led to believe that it does arise from disease about the base of the brain; but no satisfactory facts have occurred to me illustrative of this view of the subject, in addition to those which I have formerly referred to."—" I agree with you in doubting whether the chord has been sufficiently examined in those cases that have been ascribed to disease of the brain." Thus the powerful authority of Dr. Abercrombie cannot, with any propriety, be now adduced as confirming the supposed cerebral origin of paraplegia.

### II. Dr. Baillie's *Cases of Paraplegia.*

In respectfully examining the recorded data on which that excellent physician founded his opinions, I trust I shall not be chargeable with presumption. The paper which, in the southern part of our island, produced the greatest impression, in reference to the cerebral origin of paraplegia, appeared in the sixth volume of the Transactions of the London College of Physicians. In that communication, the author candidly admits that he had " not had much opportunity of becoming acquainted with the morbid appearances of the disease;" and, with his characteristic modesty, offers his remarks, in order that the opinion "may be either established or properly limited by the future observations of other practitioners."

In the commencement of the paper, Dr. Baillie states it as his opinion that " in adults, where there has been no accident affecting the spine by outward violence, paraplegia depends *most commonly* upon a disease affecting the brain itself." To illustrate this opinion, one case only is adduced, although several other examples appear to have occurred in the author's practice.

In this case, as the author remarks, " the diseased appearances of the brain were very strongly marked on dissection."

" The bones of the skull, more especially at the sutures, were more vascular than usual; the dura mater presented nearly its natural appearance, but the vessels of the pia mater were very much loaded with blood, and there were effusions of serum between the different membranes of the brain on both sides of it. The tunica arachnoides was opaque, and much thickened. The substance of the cerebrum was considerably firmer, and that of the cerebellum was considerably softer than natural. About four ounces of water were found in the lateral ventricles of the brain, and a considerable quantity of water was discharged from within the theca of the spinal marrow."*

Here, I would with deference observe, no mention is made of the state of the spinal marrow or its investing membranes. The water "discharged" from within the theca probably emerged from that enclosure, on the medulla spinalis being divided within a short distance of its commencement. We have, therefore, no *proof* that concomitant disease did not exist within the rachidian canal. We are not, indeed, informed at what period of the head-affection the paraplegic symptoms supervened, and are therefore unable, in the absence of an examination of the spine itself, to trace any extension of disease from the brain downwards. Yet, considering the change of structure which the arachnoid tunic of the brain had undergone, it is not at all improbable that the inflammation had extended to the arachnoid of the spinal chord; in which case, serous effusion within the theca may have been a direct consequence of the spinal arachnitis itself. It is not necessary, however, to rest our argument on any such probability. We have decided proof of water within the theca; *the presence of which alone, from whatever source it may have arisen, is adequate, I conceive, to account for the paraplegia, irrespective of any morbid condition of the brain.* If, therefore, the mechanical pressure of the serum upon the spinal marrow be admitted as a sufficient cause of the paraplegia, we are surely not justified in ascribing it to the state of the head.

No one will affirm, I apprehend, that the existence of so much disease in cranio will more satisfactorily account for the paraplegia, than the mere pressure of fluid within the theca. Numerous examples of still greater disease of the brain might be adduced, in which there had been no paraplegia; while, on the contrary, pressure from a fluid, or other foreign body, upon the spinal marrow, rarely exists without inducing paraplegia in a greater or less degree. The case, therefore, cannot be regarded as a conclusive instance of cerebral paraplegia. So far as this case is concerned, we may at least say, in the language of the Scottish courts, "Not proven."

In a note to the paper we have been considering, there is an allusion to the opinions of Sir Henry Halford, Sir James Earle, and Mr. Copeland, as favourable to the same view of the subject. The opinions of the two former of

* Medical Trans. vol. vi. p. 22-3. Dr. Baillie states that he received the account of this examination from Mr. Pennington.

these gentlemen were probably communicated to Dr. Baillie in conversation. I have not, at least, been able to find any recorded statement of the view of the learned Baronet on paraplegia; and consequently have not the advantage of ascertaining the data upon which the opinion alluded to by Dr. Baillie has been founded. In carefully examining, also, the editorial notes by Sir James Earle, in his edition of the late Mr. Pott's works, in reference to the palsy of the lower limbs, I have been equally unsuccessful; having met with no notice of paraplegia from disease within the head. In the excellent observations of Mr. Copeland "on the Symptoms and Treatment of the Diseased Spine," there is, however, a passage, in which that respectable author gives it as his opinion, that there are cases of palsy of the lower limbs, the cause of which is connected with the functions of the brain: but no cases are adduced to substantiate that opinion; nor is it, indeed, stated whether the opinion be drawn from the author's own observation. The cases detailed in Mr. Copeland's work are examples of disease arising from injury of the spine, and are therefore irrelevant to our present inquiry.

In reflecting on the prevalence of the opinion just considered, it may not be improper to advert, for a moment, to the astonishing influence of a great and good name in giving to an opinion, avowedly open to "further confirmation or limitation," and confessedly resting on an insufficient basis, all the weight and force of a legitimate induction from an ample number of facts. The estimable author suggested it, indeed, for additional illustration: yet such is the proneness of the human mind to repose itself upon authority, rather than exert its energies in diligent investigation, that the hypothesis —for, without an examination of the spine, it is but an hypothesis,—has been currently received with all the influential confidence of a well-established fact.*

I now beg to introduce the interesting document, by the late Dr. Baillie, which Dr. Gregory was so obliging as to put into my hands on the evening of the discussion before referred to, having first read it to the Society. It is folded up in the form of a letter, and marked at the back "Short Memoranda of Cases of Paraplegia." I give it verbatim.

* In proof of the implicit admission of this doctrine, I may extract a sentence from the last edition of Dr. Mason Good's Study of Medicine, in which the lamented author, notwithstanding the vast extent of his medical and various erudition, affirms that "The best practical writers of the present day concur in opinion that paraplegia, like hemiplegia, is produced still more frequently by causes operating on the brain, than confined to the spine. Of this opinion is Dr. Baillie," &c. &c. (Vol. iv. p. 666.)

"FACTS RELATIVE TO PARAPLEGIA,

(*From a posthumous MS. of the late* Dr. Baillie.)

"A clergyman had gutta serena of one eye along with paraplegia.

"A nobleman had the vision of both eyes very much impaired in paraplegia from gutta serena, but this affection at length a good deal subsided.

"A gentleman had a temporary gutta serena, and an occasional dropping of one eyelid, with paraplegia.

"A gentleman had his memory much impaired, and his mind so confused, that he could not keep his own little domestic accounts, during the latter period of paraplegia.

"A gentleman had a dilatation of the pupil of the right eye, with an occasional dropping of each upper eyelid, in paraplegia. The dropping of the eyelids has subsided.

"Mr. Earle told me that he had attended a case of paraplegia, in which the intellect was extremely imperfect for a considerable time before the patient's death. Tumors were found, upon dissection, to be formed in the brain.

"A young lady was subject to very severe headaches, to considerable drowsiness, and occasional defect of memory, in paraplegia.

"A young man had double vision for more than six weeks in paraplegia. His arms were numb and weak, and he had sometimes great difficulty in writing.

"A lady had severe headaches, and numbness and weakness in her hands, in paraplegia.

"A gentleman had great weakness and numbness of his hands in paraplegia, so that he could not distinguish, by his feeling, a shilling and a sixpence from each other.

"Another gentleman, in paraplegia, had gutta serena of his left eye; had great weakness in his arms, with indistinct feeling, so that he said he could not distinguish, by the touch, shillings and sixpences from each other.

"A lady, in paraplegia, had impaired vision, severe headaches, and weakness in her arms and hands, so that often objects that she held in her hands would drop from them.

"A gentleman, in paraplegia, had much giddiness of the head, and could write (from the weakness of his hands) with great difficulty.

"A gentleman, in paraplegia, had great confusion of the head, occasional defect of memory, occasional paralytic imperfection of speaking, and often wrote very indistinctly. His hand-writing formerly was remarkably distinct.

"A gentleman, in paraplegia, had severe headache, the right eye blind, the pupil of the left eye a good deal dilated, and a memory sometimes defective.

(Signed) "M. B."

"*Dec.* 8*th*, 1822."

In presuming to offer any comment on these "Short Memoranda," I feel that I am treading on sacred ground. The very existence of such memoranda, in reference to so considerable a number of facts, clearly evinces the great attention which the venerated writer continued to pay to the subject of paraplegia. Brief as the notices are, there can be no doubt but that, on Dr. Baillie's own perusal, they revived in his mind a full and satisfactory impression of the cases to which they refer, and afforded a confirmation of his opinion that paraplegia often arises from cerebral affection. They certainly prove the frequent *coincidence* of affections of the head with paraplegia; but, as evidences of head-affection and paraplegia bearing the relation of cause and effect to each other, they must be regarded, in my humble opinion, as altogether inconclusive. The spine does not appear to have been examined: we have no proof, therefore, that the spinal marrow, or its meninges, were not also diseased. It may, indeed, be presumed, from a knowledge of Dr. Baillie's extent and accuracy of observation, that had any symptoms during life denoted disease within the spinal column, such symptoms would have been recognised and recorded; and that, in the absence of any such reference in the Doctor's Memoranda, we ought to conclude that no such symptoms existed. And, most assuredly, this is a fair presumption, so far as the more obvious characters of spinal disease are concerned. It is possible, however, that some of the less evident affections of the spinal marrow, and such particularly as have been more recently investigated by the continental pathologists, may have escaped even the penetrating view of that very able physician; especially with a mind prepossessed in favour of the cerebral origin of paraplegia, and when occupied with cases exhibiting such undoubted evidence of cerebral disease. But allowing that every examination was made which, during the life of the patient, was possible, and that no disease of the spine could be detected, the same objection which has been urged against the cerebral origin of Dr. Baillie's published cases, would be also applicable to these,—viz. that fluid may have descended into the vertebral theca, and, by its pressure on the spinal marrow, have produced the paraplegia.

While, however, we may be unable to receive these brief notices as conclusive proofs of the cerebral origin of paraplegia, we are ready to admit that the hypothesis which they uphold carries with it an air of considerable probability, but which an examination of the spine, as well as of the brain, in future examples of a similar kind, can alone verify or confute. In a practical point of view, these posthumous contributions ought, notwithstanding, to be received with grateful acknowledgment; inasmuch as they direct the attention of the physician to the intimate connexion which subsists between morbid conditions of the brain and morbid conditions of the spine; and the absolute necessity of carefully attending to every cerebral symptom in the treatment of paraplegia. In truth, both the brain and the spinal marrow should be vigilantly watched, and every morbid symptom in either part strenuously opposed, in our endeavours to remove the disease. Exclusive attention to the one, whatever may be our view of the proximate cause of paraplegia, can only lead to error and disappointment.

Agreeably to the plan proposed in the commencement of this paper, I now proceed to consider the opinions of some other eminent pathologists on the same subject. The length to which the preceding observations have already extended, will, however, only permit me to advert to these authorities very briefly. The number of cases possessing *any* claim to be considered as examples of "cerebral" paraplegia, is not indeed considerable; and of that number, by far the largest proportion is liable to the same objection which has been urged against the cases already considered,—viz. that the spine was not examined; the morbid condition of the brain having been gratuitously regarded as fully adequate to the production of the paraplegia. Indeed, the state of the spine has been very rarely investigated in those cases which had exhibited strong characters of head-affection; and hence our illustrations are necessarily far less numerous than might have been supposed. Even the late Dr. Parry, that distinguished pathologist and philosopher, at the close of a life ardently devoted to medical science, confesses that only "one instance of investigating by dissection the cause of paraplegia" had occurred to him. To that instance we shall presently refer.

The invaluable work of Morgagni yields but little specific information on our present subject. One example recorded by that indefatigable pathologist has already been cited. In another instance of paraplegia which followed hemiplegia, the author found an accumulation of serum between the dura and pia mater; but he is altogether silent with respect to the spine. It is highly probable, from the accumulation of serum, as well as from the fact of the paraplegia having succeeded to the hemiplegia, that, in this case also, the fluid insinuated itself into the theca vertebralis, and thus gave rise to the paraplegic symptoms.

In the admirable collection of BONETUS, I have been equally unable to obtain any satisfactory examples of "cerebral" paraplegia. In the fifteenth section of his first Book, he has indeed given us, with his accustomed conciseness, the post-mortem appearances of an example of paraplegia occurring "in phrenitico, ob serum multum in ventriculo medio deprehensum, cum siccitate medullæ cerebri et membranorum." But, as no mention is made of the spinal canal, and as, in all probability, the serum, by gravitating into the vertebral theca, had an important influence in producing the paraplegia, no proof of its merely cerebral origin can be drawn from such a statement.

In the "Collections from the unpublished Medical Writings" of Dr. PARRY, is the result of that single opportunity of investigating by dissection the cause of paraplegia, to which we lately alluded. "In that, the membrane investing the spinal marrow, as far as consistently with a due regard to appearances it could be traced, was almost every where of a deep red colour, from excessive vascularity."—"The patient to whom I allude," says Dr. Parry, "had the affection first in the lower limbs, and afterwards in the hands also; finally, he died phrenitic. Conformably to this latter symptom, the pia mater was found in a state of inflammation."* (Vol. i. p. 510.)

This is an important fact, not only as proving the coexistence of disease in the brain and spinal marrow, but also as exhibiting, in the progress of the case, that extension of disease from the spine to the head which sometimes occurs, especially when the tunica arachnoides is the principal seat of inflammation. If the spine had not been subjected to the test of examination, an ordinary observer, forming his opinion of the case chiefly from the predominating affection of the brain, which occurred during its latter stages, would probably have ascribed the paraplegia to the state of the head alone; so important is it to ascertain the exact order and succession of symptoms in the more complicated forms of disease.

* Candour requires me to add, that Dr. Parry believed in the probability of paraplegia sometimes arising from cerebral causes, although his philosophic mind did not allow itself to rest on such a mere probability.

The researches of the French pathologists, which have contributed such ample stores to our previous information relative to the morbid conditions of the brain and spinal marrow, do not seem to have been specially directed to the elucidation of our present subject; yet, incidentally, they afford very interesting illustrations on some collateral points.

In a recent work, by le Docteur Bouillaud, of Paris, entitled "Traité Clinique et Physiologique de l'Encéphalite et des Suites," a few examples of morbid changes of structure are given as connected *with* paraplegia; but generally without any reference to the state of the spine. One of the cases, in which we have to lament the same deficiency, exhibited, on dissection, considerable effusion into both the lateral ventricles, with marks of high inflammation in the surrounding cerebral substance. In the absence, therefore, of any evidence respecting the state of the spine, it is not unfair to conjecture that the fluid from the ventricles had made its way into the theca, and thus compressed the spinal cord.

The case of Marie Machelein, cited by M. Bouillaud, is not a little important, as showing *the concomitance of cerebral and spinal disease.* The patient had left the hospital (des Enfans) "almost cured" of a hemiplegia of the right side. Four months after, weakness of the lower limbs supervened, and soon increased to perfect paraplegia. Motion was first lost; then sensation. At length, respiration became affected. In the progress of the disease, the arm originally paralysed became still weaker. *On dissection,* that portion of the left hemisphere which forms the roof of the lateral ventricle was found so indurated as to resist the scalpel. There was effusion of coagulated blood within the vertebral canal; the spinal marrow itself was completely disorganised, and reduced to a kind of reddish "bouillie." (P. 187-8.)

Here we have a striking exemplification of the necessity of examining the spine as well as the head, in order to form a correct judgment of the real cause of paraplegia. Had not the state of the spinal marrow been investigated, the mere fact of the paraplegia having followed the hemiplegia might have strengthened the supposition of the former, as well as the latter, having its origin within the head.

Still further to illustrate the co-existence of disease in the brain and spinal marrow, and to which coincidence, in all probability, several of the alleged cases of "cerebral" paraplegia must have been referred, if the spine as well as the head had been duly examined,—I shall now introduce a case from the masterly work of le Docteur Ollivier, "De la Moëlle Epinière, et de ses Maladies," (à Paris, 1824.)

Margaret Marshall, æt. seventy-nine, had laboured under pulmonary catarrh, and violent pain of the head, but without any general disturbance of the cerebral functions. She then experienced a sense of formication, followed by a diminution, and at length an entire loss, of feeling in one arm and leg. Ultimately, there was complete paraplegia. Upon dissection, the membranes of the brain were found to be infiltrated with serum. There was also a general mollescence ("ramolissement") of the brain, and of the whole diameter of the spinal marrow, to the extent of an inch and a half, in its cervical portion.—May we not presume that, in this case also, if the state of the spinal cord had been overlooked, the paraplegic symptoms would have been solely attributed to a cerebral cause?

In another example from the same incomparable treatise, notwithstanding the degree of cerebral mischief which it exhibits, the paraplegia may probably have arisen from the concomitant disease of the spinal marrow. I adduce it as an additional argument against confiding in the alleged cases of "cerebral" paraplegia; and as also furnishing an additional motive for investigating both the spine and the head in the future instances of the disease.

Louis Spréval had been observed to be remarkably indolent for several years. He walked with an unsteady gait, and was sometimes maniacal. At the end of nine years, the power of motion was lost in the lower extremities, which retained, notwithstanding, their sensibility. On dissection, the cranium was found to be of thrice its ordinary thickness. The dura mater was thickened, as was also the pia mater covering the pons Varolii and the corpora olivaria. The pia mater investing the corpora olivaria et pyramidalia being raised, these bodies were found in a pulpy state. *The anterior part of the spinal marrow exhibited a similar condition.*

The inquiry has hitherto been confined to published documents. I have not been, however, inattentive to the opinions of those among my brethren, to whom I had access, whose pathological knowledge might enable them to throw light upon the subject; and although I should not feel myself justified, without express permission, in specifying names, there can be no objection to my stating the general result of such conversational inquiries. Of twelve physicians and hospital surgeons, who kindly favoured me with their sentiments, the greater number candidly admitted that they had not particularly investigated the subject, but had rather taken the opinion for granted, as proceeding from such high authority. Two of these gentlemen informed me that they had long been dissatisfied with the doctrine. All, with two exceptions, acknowledged that no opportunity of verifying the opinion, by an examination of the spine itself, had yet occurred to them. The respectable individuals forming the two exceptions were, however, not less distinguished than the rest for pathological research. One of them informed me that he had carefully inspected the spinal column, in many examples of aged females who had died with symptoms of paraplegia, without finding within it any trace of disease; the morbid appearances having been confined to the head alone, and consisting chiefly of serous effusion under the tunica arachnoides, and into the lateral ventricles.

This communication appeared to me of so much importance, that I ventured to request further particulars; but have not hitherto been so fortunate as to receive them.

The other gentleman, whose opinion likewise favoured the cerebral origin of paraplegia, assured me that he had seen several cases of paraplegia, similar to those described by Dr. Baillie, in which the brain alone was affected, and the spine, though carefully examined, found perfectly free from disease.

These statements are undoubtedly of considerable importance. The object of this paper being to elicit truth rather than to subvert an opinion, I shall feel highly gratified if this general notice of the conversations alluded to should induce the two gentlemen concerned to favour the public with a more particular account of those cases and dissections, should they have been the subject of record. I am well aware of the difficulty of preserving an exact recollection of morbid appearances long after they have been observed: and I am not insensible to the important influence of circumstances, apparently trivial, in the history and description of the structural changes induced by disease. I shall therefore anxiously wait for that additional confirmation which a reference to the written memoranda of these cases may afford.

Notwithstanding, however, these two opposing statements, the conclusions fairly deducible from the *recorded* examples of the disease remain unaffected, and may be thus expressed:

1st. From the cases of paraplegia examined in this paper, as well as from every other recorded example which the writer has been able to obtain, no proof whatever can be adduced of the disease having arisen from cerebral causes alone.

2dly. From the history and symptoms of several of the alleged examples of paraplegia, it is more probable, in the absence of direct proof, that concomitant disease existed within the spinal column, than that the brain alone had suffered from morbid changes.

3dly. In some other of the alleged examples, in which fluid was discharged from the vertebral theca, we have no proof but that the mere presence of the fluid within the theca, independently of the cause which produced it, may have occasioned the paraplegia.

To these general inferences, I might adduce several unquestionable examples of co-existing disease in the brain and in the spinal marrow; and might also prove, by recorded facts, the ultimate extension of disease from the spinal cord to the brain, during the progress of paraplegiæ which had commenced in the spine, and had long remained without any cerebral affection whatever. I might also prove, from incontrovertible premises, that true spinal paraplegia has sometimes been attended with symptoms indicative of slight cerebral affection, in cases which, after death, exhibited no change of structure within the cranium, but great disorganisation of the spinal marrow itself. Such evidence would be chiefly valuable as showing the high importance of carefully attending to the spinal, as well as the cerebral, symptoms, of the disease; and of not hastily concluding that the paraplegia arises from a morbid condition of the brain, merely because it is attended with some degree of cerebral disturbance. But the proper limits of this paper having already been exceeded, I must reserve these materials, as well as some collateral facts which have fallen under my own observation, for another opportunity. Should life and health permit, I may hope, on some future occasion, to submit them to the profession, together with the results of some other inquiries connected with the diseases of the brain and spinal chord.

Before taking leave of the subject, I must beg, however, explicitly to state that, strongly as I contend for the verdict of "Not proven," in relation to the evidence hitherto recorded in favour of the "cerebral" origin of paraplegia, I am by no means prepared to argue against the *possibility* of its proceeding from cerebral causes alone. On the contrary, from the connexion known to subsist between certain lesions of the brain and certain paralytic affections, I am only surprised that indubitable proof of such an origin has not already been established. It is well known that certain morbid changes occurring in a small portion of one hemisphere of the brain, have induced a paralysis of one leg; and it is equally true that a more extensive lesion of one hemisphere has occasioned a palsy of one entire half of the body. Now it is very conceivable, that a degree of cerebral injury greater than the former, yet less than the latter, may occur; and that the paralytic affection thereby induced, instead of amounting to a perfect hemiplegia, may merely include the leg, thigh, and corresponding half of the pelvis. Pursuing the supposition still further: should it ever happen that the same extent of injury takes place, at the same time, in both hemispheres, we should then have precisely that condition of brain from which we might expect the production of paraplegia. I am fully aware that, most commonly, one hemisphere only is affected; and that, hence, hemiplegia is by far the most prevalent form of paralysis. I am also aware that, when both hemispheres are extensively injured, universal palsy is the general result: but we may readily admit the possibility of a degree of disease in both hemispheres, short of that which would have produced universal palsy, yet sufficient to paralyse the lower half of the body, or a more considerable portion of it, taken transversely. Dr. Baillie himself thought it probable that paraplegia, when produced by disease within the head, arose from a morbid condition of both hemispheres; but cautiously proposed the subject for future investigation. Difficulties, indeed, meet us at every step. The diseases of the nervous system continue to be involved in peculiar perplexity. Cases of paraplegia have lately occurred to my distinguished friend Dr. Abercrombie, in which no morbid change whatever could be detected either in the brain or spinal marrow! Yet let us not despair. The path may indeed be rugged, and our progress slow and interrupted, but doubtless some good will be achieved; while, in the mean time, the very effort is useful, and brings with it its own reward.

*Great Ormond-street; April 10th, 1827.*

